# Supplementary material for: Deciphering HLA-I motifs across HLA peptidomes improves neo-antigen predictions and identifies allostery regulating HLA specificity
Source: PLoS Comput Biol. 2017 Aug 23;13(8):e1005725. doi: 10.1371/journal.pcbi.1005725 (PMC5584980; doi:10.1371/journal.pcbi.1005725)
Supplement: S1 Supporting Information — (PDF) [file pcbi.1005725.s001.pdf]

Supplementary Information

Deciphering HLA-I motifs across HLA peptidomes improves neo-antigen predictions and identifies allostery regulating HLA specificity

Michal Bassani-Sternberg[1,2,*], Chloé Chong[1,2], Philippe Guillaume[1,2], Marthe Solleder[1,3], HuiSong Pak[1,2], Philippe O Gannon[2], Lana E Kandalaft[1,2], George Coukos[1,2], David Gfeller[1,2,3,*]

[1]Ludwig Centre for Cancer Research, University of Lausanne, 1066 Epalinges, Switzerland.

[2]Department of Fundamental Oncology, University Hospital of Lausanne, Lausanne, Switzerland.

[3]Swiss Institute of Bioinformatics (SIB), 1015 Lausanne, Switzerland.

[*]To whom correspondence should be sent: David.Gfeller@unil.ch and Michal.Bassani@chuv.ch.

Supplementary Figures

Mel_15

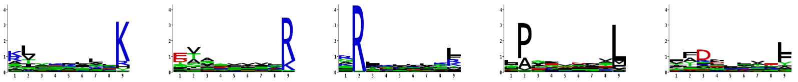

HLA-A03:01 - 2242 HLA-A68:01 - 3502 HLA-B27:05 - 2441 HLA-B35:03 - 2610 HLA-C04:01 - 299

Mel_16

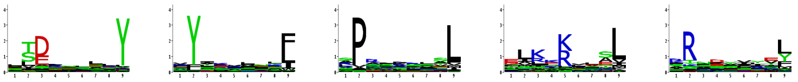

HLA-A01:01 - 1085 HLA-A24:02 - 1463 HLA-B07:02 - 2292 HLA-B08:01 - 2311 Not assigned - 309

Mel_12

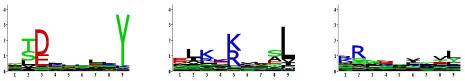

HLA-A01:01 - 575 HLA-B08:01 - 1765 HLA-C07:01 - 127

Mel_8

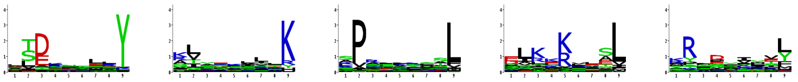

HLA-A01:01 - 567 HLA-A03:01 - 878 HLA-B07:02 - 1300 HLA-B08:01 - 539 HLA-C07:01 - 124

Mel_5

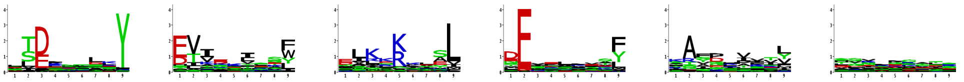

HLA-A01:01 - 353 HLA-A25:01 - 628 HLA-B08:01 - 1074 HLA-B18:01 - 693 Not assigned - 233 Not assigned - 127

Fibroblast

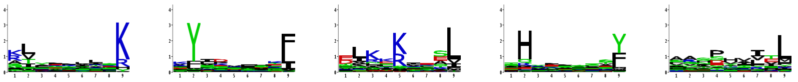

HLA-A03:01 - 1249 HLA-A23:01 - 752 HLA-B08:01 - 402 HLA-B15:18 - 902 HLA-C07:04 - 187

HCC1143

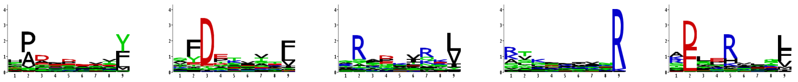

HLA-B35:08 - 425 HLA-C04:01 - 87 HLA-C06:02 - 347 Not assigned - 762 Not assigned - 368

HCC1937

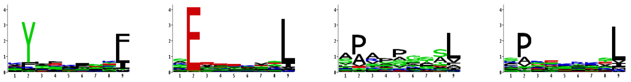

HLA-A23:01 - 908 HLA-B40:01 - 1032 Not assigned - 562 Not assigned - 424

HCT116

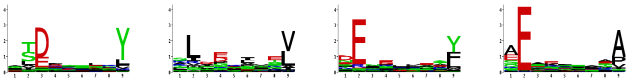

HLA-A01:01 - 614 HLA-A02:01 - 606 HLA-B18:01 - 347 HLA-B45:01 - 1098

JY

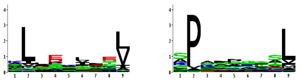

HLA-A02:01 - 897 HLA-B07:02 - 843

Bcell

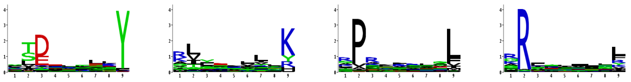

HLA-A01:01 - 549 HLA-A03:01 - 990 HLA-B07:02 - 2339 HLA-B27:05 - 2096

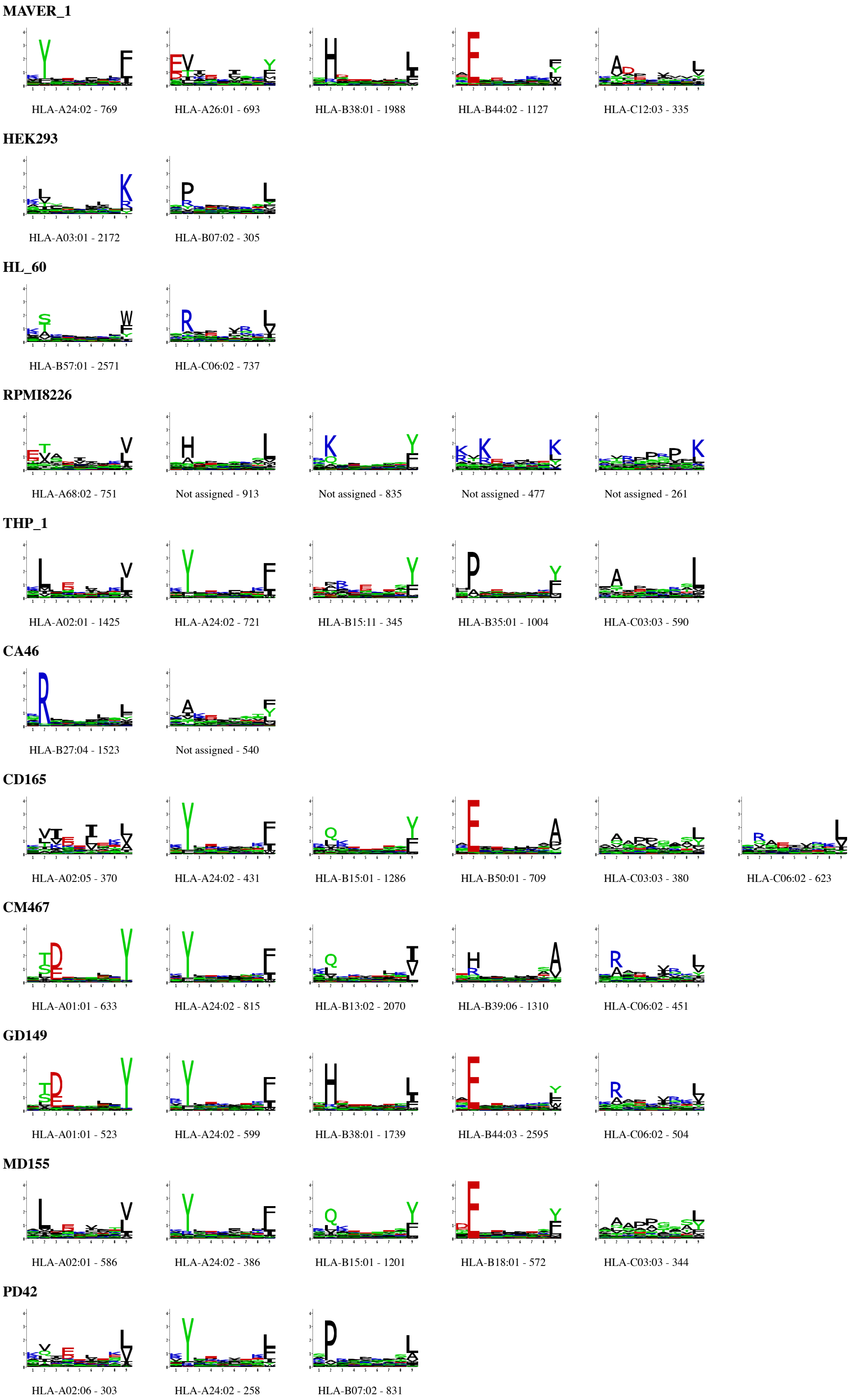
MAVER_1
HLA-A24:02 - 769
HLA-A26:01 - 693
HLA-B38:01 - 1988
HLA-B44:02 - 1127
HLA-C12:03 - 335
HEK293
HLA-A03:01 - 2172
HLA-B07:02 - 305
HL_60
HLA-B57:01 - 2571
HLA-C06:02 - 737
RPMI8226
HLA-A68:02 - 751
Not assigned - 913
Not assigned - 835
Not assigned - 477
Not assigned - 261
THP_1
HLA-A02:01 - 1425
HLA-A24:02 - 721
HLA-B15:11 - 345
HLA-B35:01 - 1004
HLA-C03:03 - 590
CA46
HLA-B27:04 - 1523
Not assigned - 540
CD165
HLA-A02:05 - 370
HLA-A24:02 - 431
HLA-B15:01 - 1286
HLA-B50:01 - 709
HLA-C03:03 - 380
HLA-C06:02 - 623
CM467
HLA-A01:01 - 633
HLA-A24:02 - 815
HLA-B13:02 - 2070
HLA-B39:06 - 1310
HLA-C06:02 - 451
GD149
HLA-A01:01 - 523
HLA-A24:02 - 599
HLA-B38:01 - 1739
HLA-B44:03 - 2595
HLA-C06:02 - 504
MD155
HLA-A02:01 - 586
HLA-A24:02 - 386
HLA-B15:01 - 1201
HLA-B18:01 - 572
HLA-C03:03 - 344
PD42
HLA-A02:06 - 303
HLA-A24:02 - 258
HLA-B07:02 - 831

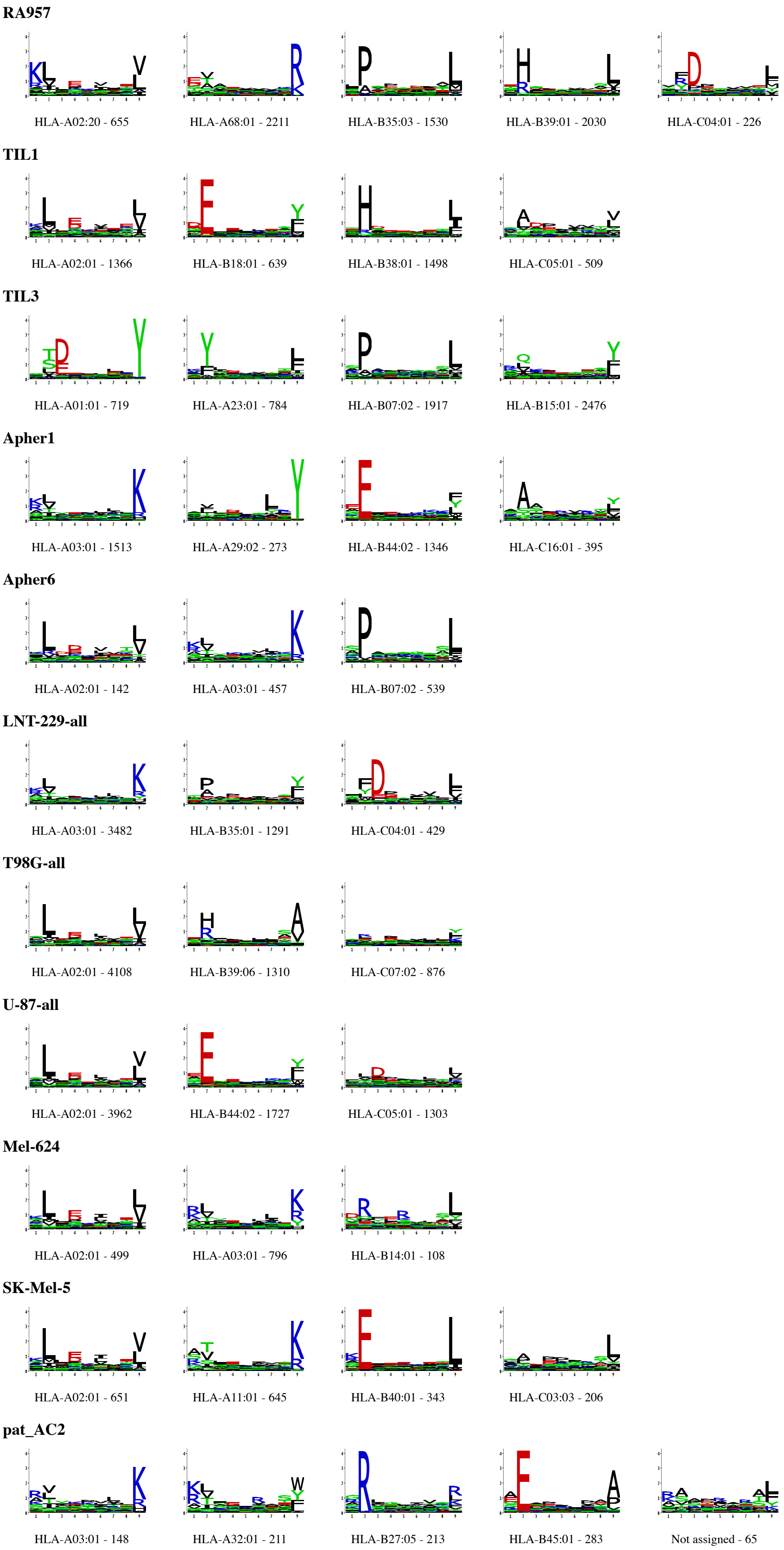
RA957
HLA-A02:20 - 655
HLA-A68:01 - 2211
HLA-B35:03 - 1530
HLA-B39:01 - 2030
HLA-C04:01 - 226
TIL1
HLA-A02:01 - 1366
HLA-B18:01 - 639
HLA-B38:01 - 1498
HLA-C05:01 - 509
TIL3
HLA-A01:01 - 719
HLA-A23:01 - 784
HLA-B07:02 - 1917
HLA-B15:01 - 2476
Apher1
HLA-A03:01 - 1513
HLA-A29:02 - 273
HLA-B44:02 - 1346
HLA-C16:01 - 395
Apher6
HLA-A02:01 - 142
HLA-A03:01 - 457
HLA-B07:02 - 539
LNT-229-all
HLA-A03:01 - 3482
HLA-B35:01 - 1291
HLA-C04:01 - 429
T98G-all
HLA-A02:01 - 4108
HLA-B39:06 - 1310
HLA-C07:02 - 876
U-87-all
HLA-A02:01 - 3962
HLA-B44:02 - 1727
HLA-C05:01 - 1303
Mel-624
HLA-A02:01 - 499
HLA-A03:01 - 796
HLA-B14:01 - 108
SK-Mel-5
HLA-A02:01 - 651
HLA-A11:01 - 645
HLA-B40:01 - 343
HLA-C03:03 - 206
pat_AC2
HLA-A03:01 - 148
HLA-A32:01 - 211
HLA-B27:05 - 213
HLA-B45:01 - 283
Not assigned - 65

pat_C

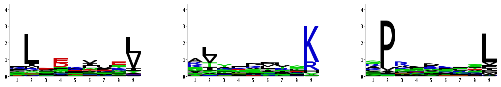

HLA-A02:01 - 224 HLA-A03:01 - 243 HLA-B07:02 - 956

pat_CELG

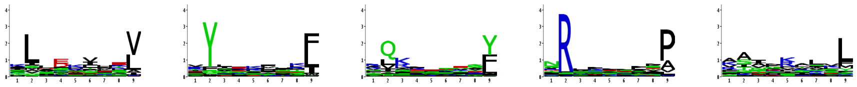

HLA-A02:01 - 366 HLA-A24:02 - 360 HLA-B15:01 - 721 HLA-B73:01 - 456 HLA-C03:03 - 165

pat_CP2

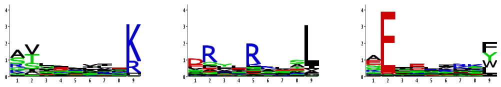

HLA-A11:01 - 364 HLA-B14:02 - 350 HLA-B44:02 - 341

pat_FL

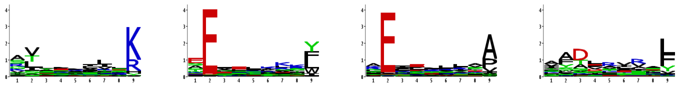

HLA-A03:01 - 843 HLA-B44:03 - 706 HLA-B50:01 - 442 Not assigned - 136

pat_J

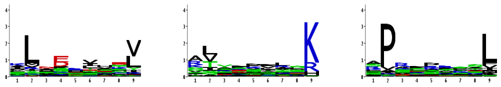

HLA-A02:01 - 173 HLA-A03:01 - 234 HLA-B07:02 - 808

pat_JPB3

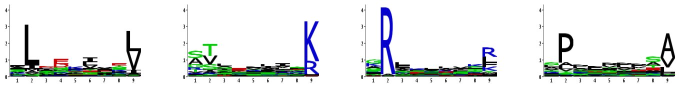

HLA-A02:01 - 302 HLA-A11:01 - 237 HLA-B27:05 - 283 HLA-B56:01 - 187

pat_JT2

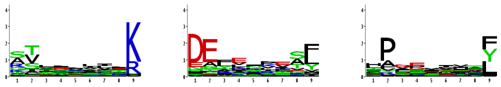

HLA-A11:01 - 498 HLA-B18:03 - 119 HLA-B35:01 - 229

pat_M

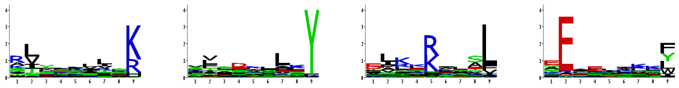

HLA-A03:01 - 274 HLA-A29:02 - 163 HLA-B08:01 - 411 HLA-B44:03 - 675

pat_MA

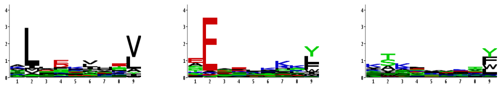

HLA-A02:01 - 430 HLA-B44:03 - 907 HLA-B57:01 - 661

pat_ML

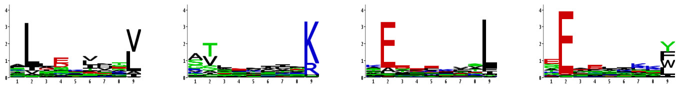

HLA-A02:01 - 268 HLA-A11:01 - 295 HLA-B40:01 - 469 HLA-B44:03 - 548

pat_NS2

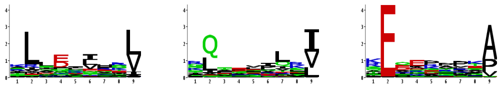

HLA-A02:01 - 205 HLA-B13:02 - 129 HLA-B41:01 - 101

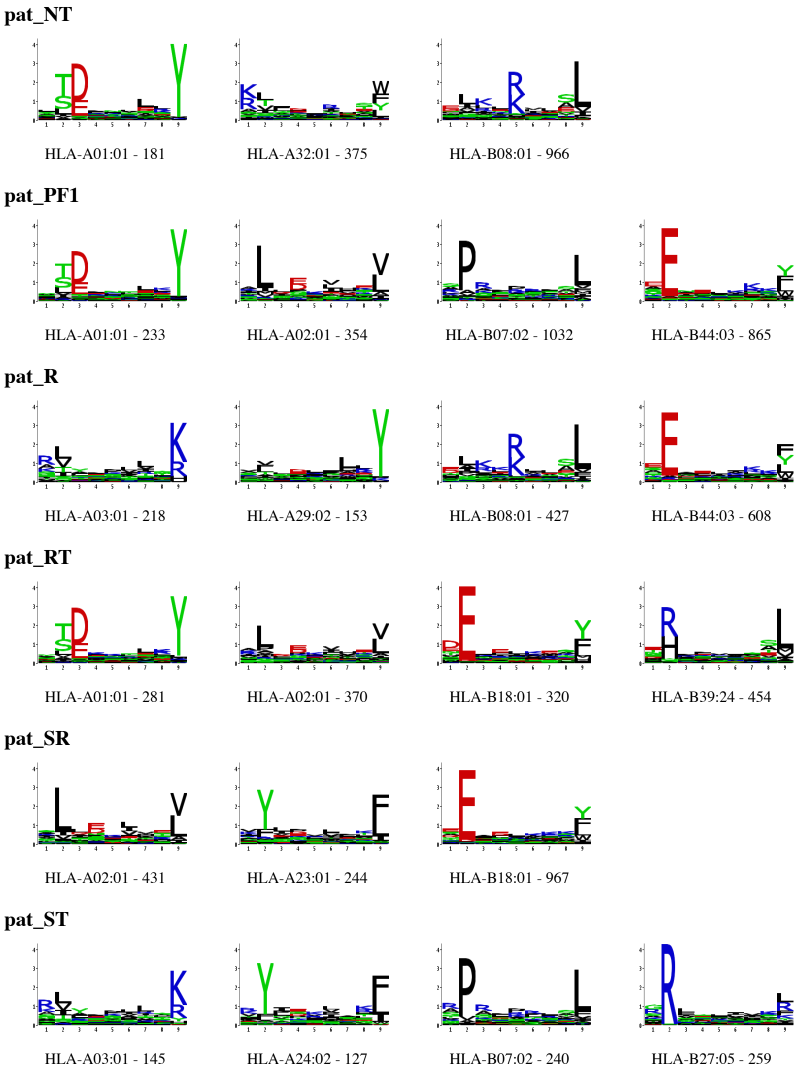

Fig A: Motifs identified across all HLA peptidomics datasets analyzed in this work. The allele to which the logos have been mapped by the approach presented in this work and the number of peptides assigned to this allele is indicated below each motif. The few motifs that could not be assigned to an allele are annotated as “Not assigned”.

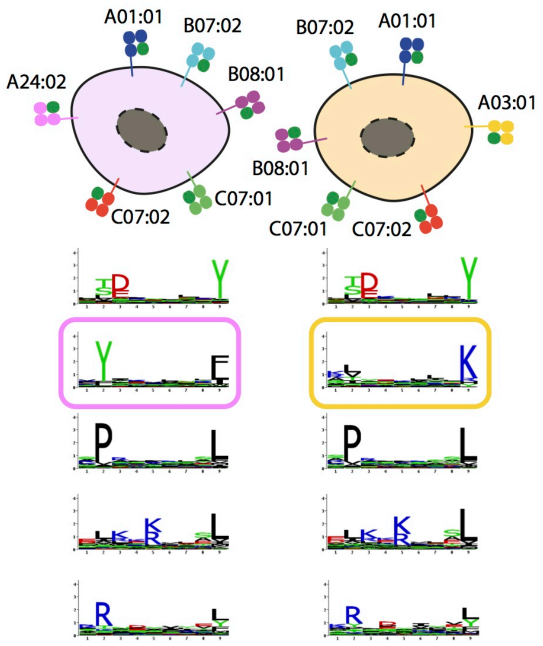

Fig B: Strategy to annotate motifs in cases where two samples share all but one motif. The left sample (Mel_16) and the right sample (Mel_8) come from two different melanoma patients from [1].

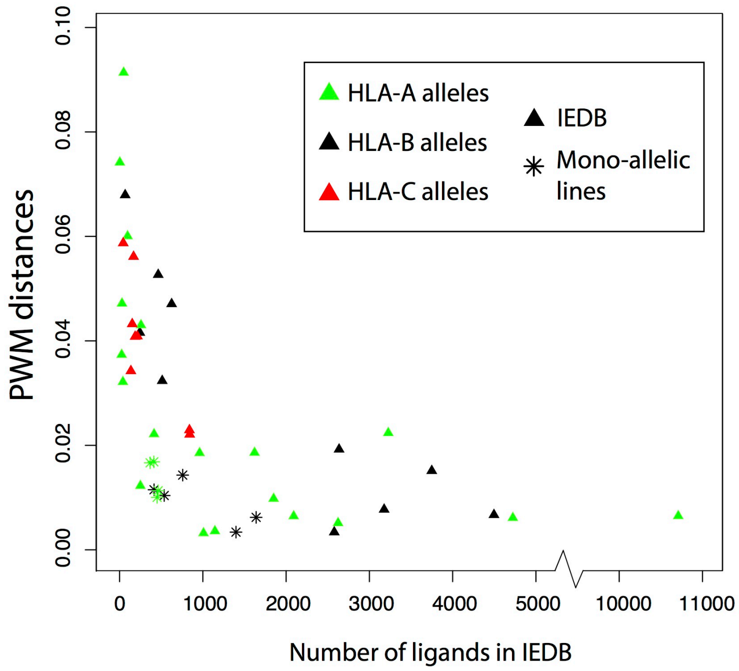

Fig C: PWM distances between the motif predicted in this work and motifs derived from IEDB, as a function of the number of ligands in IEDB.

HLA alleles	predicted motifs	MS IEDB-derived motifs	non-MS IEDB-derived motifs
A01:01*	2076 peptides	1591 peptides	845 peptides
A02:01	6886 peptides	5018 peptides	4808 peptides
A02:05	370 peptides	209 peptides	28 peptides
A02:06	303 peptides	no ligand	1388 peptides
A02:20	655 peptides	no ligand	no ligand
A03:01*	6581 peptides	955 peptides	2098 peptides
A11:01*	1072 peptides	335 peptides	2187 peptides
A23:01	1800 peptides	TFLPFIHTI	510 peptides

HLA alleles	predicted motifs	MS IEDB-derived motifs	non-MS IEDB-derived motifs
A24:02*	2344 peptides	1939 peptides	1606 peptides
A25:01	628 peptides	no ligand	65 peptides
A26:01	693 peptides	no ligand	606 peptides
A29:02*	396 peptides	3888 peptides	572 peptides
A32:01	441 peptides	2 peptides	462 peptides
A68:01	4293 peptides	12 peptides	1110 peptides
A68:02	751 peptides	24 peptides	1199 peptides

HLA alleles	predicted motifs	MS IEDB-derived motifs	non-MS IEDB-derived motifs
B07:02*	5808 peptides	3110 peptides	1403 peptides
B08:01	3460 peptides	633 peptides	975 peptides
B13:02	2106 peptides	no ligand	no ligand
B14:01	108 peptides	no ligand	no ligand
B14:02	350 peptides	17 peptides	70 peptides
B15:01*	3642 peptides	1012 peptides	2105 peptides
B15:11	345 peptides	no ligand	no ligand
B15:18	902 peptides	no ligand	no ligand
B18:01	2048 peptides	88 peptides	317 peptides
B18:03	119 peptides	no ligand	no ligand
B27:04	1523 peptides	734 peptides	23 peptides
B27:05*	3578 peptides	2083 peptides	520 peptides
B35:01*	2126 peptides	445 peptides	1220 peptides
B35:03	3106 peptides	102 peptides	HPNIEEVAL

HLA alleles	predicted motifs	MS IEDB-derived motifs	non-MS IEDB-derived motifs
B35:08	425 peptides	101 peptides	IPSIDVHHY
B38:01	2987 peptides	4 peptides	21 peptides
B39:01	2030 peptides	3 peptides	327 peptides
B39:06	2080 peptides	21 peptides	3 peptides
B39:24	454 peptides	no ligand	no ligand
B40:01*	1441 peptides	277 peptides	638 peptides
B41:01	101 peptides	40 peptides	no ligand
B44:02*	3066 peptides	1756 peptides	319 peptides
B44:03*	3367 peptides	885 peptides	253 peptides
B45:01	1250 peptides	94 peptides	123 peptides
B50:01	918 peptides	250 peptides	no ligand
B56:01	187 peptides	3 peptides	no ligand
B57:01*	2947 peptides	575 peptides	427 peptides
B73:01	456 peptides	no ligand	76 peptides

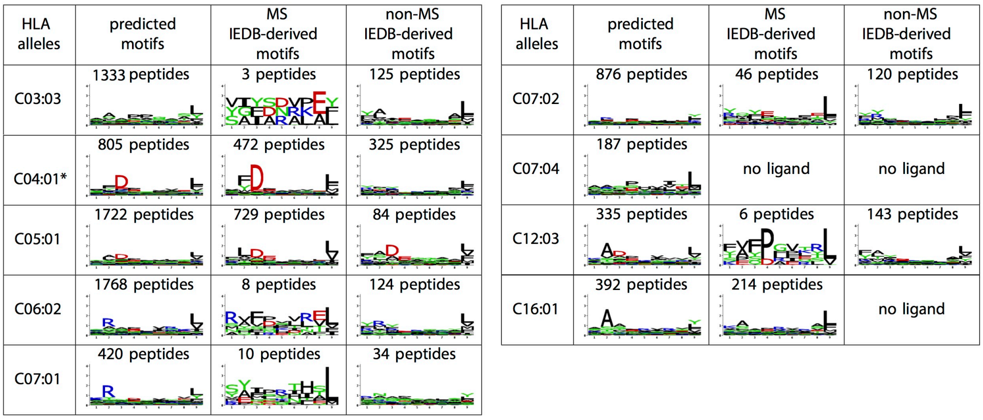

Fig D: Comparison between the HLA-I motifs identified by integrating data across all HLA peptidomics studies and the motifs from IEDB (either MS and non-MS data). When only one peptide was present the sequence of the peptide is indicated. Stars indicate motifs considered in the analyses of Fig J.

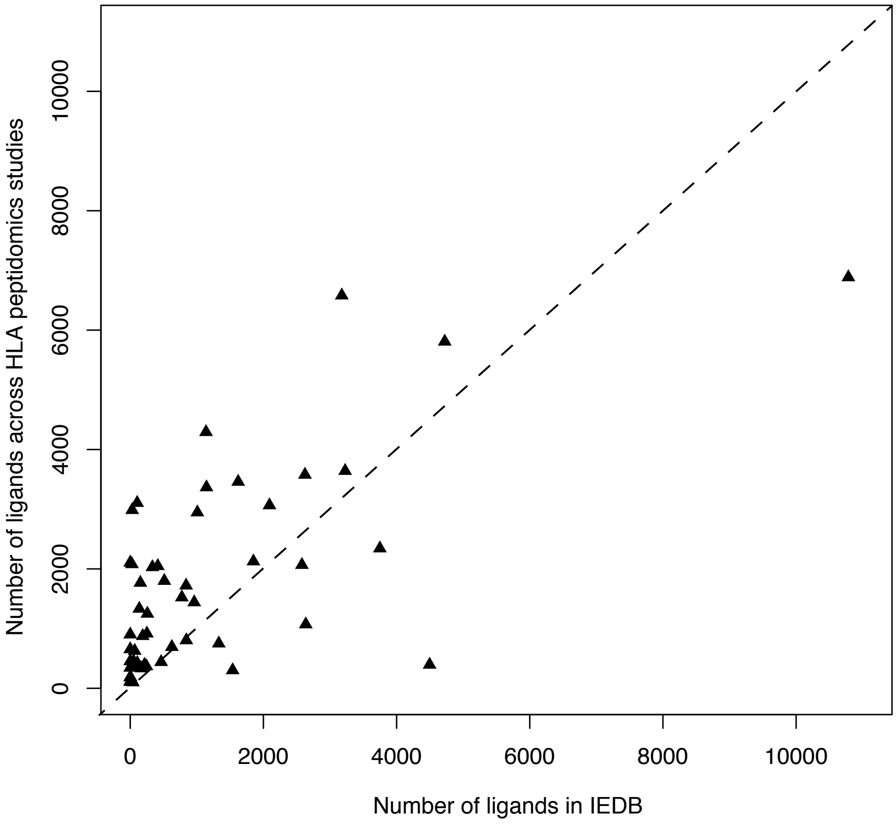

Fig E: Number of peptides assigned to each allele across the HLA peptidomics studies considered in this work, compared to IEDB data (see all raw numbers in Fig D).

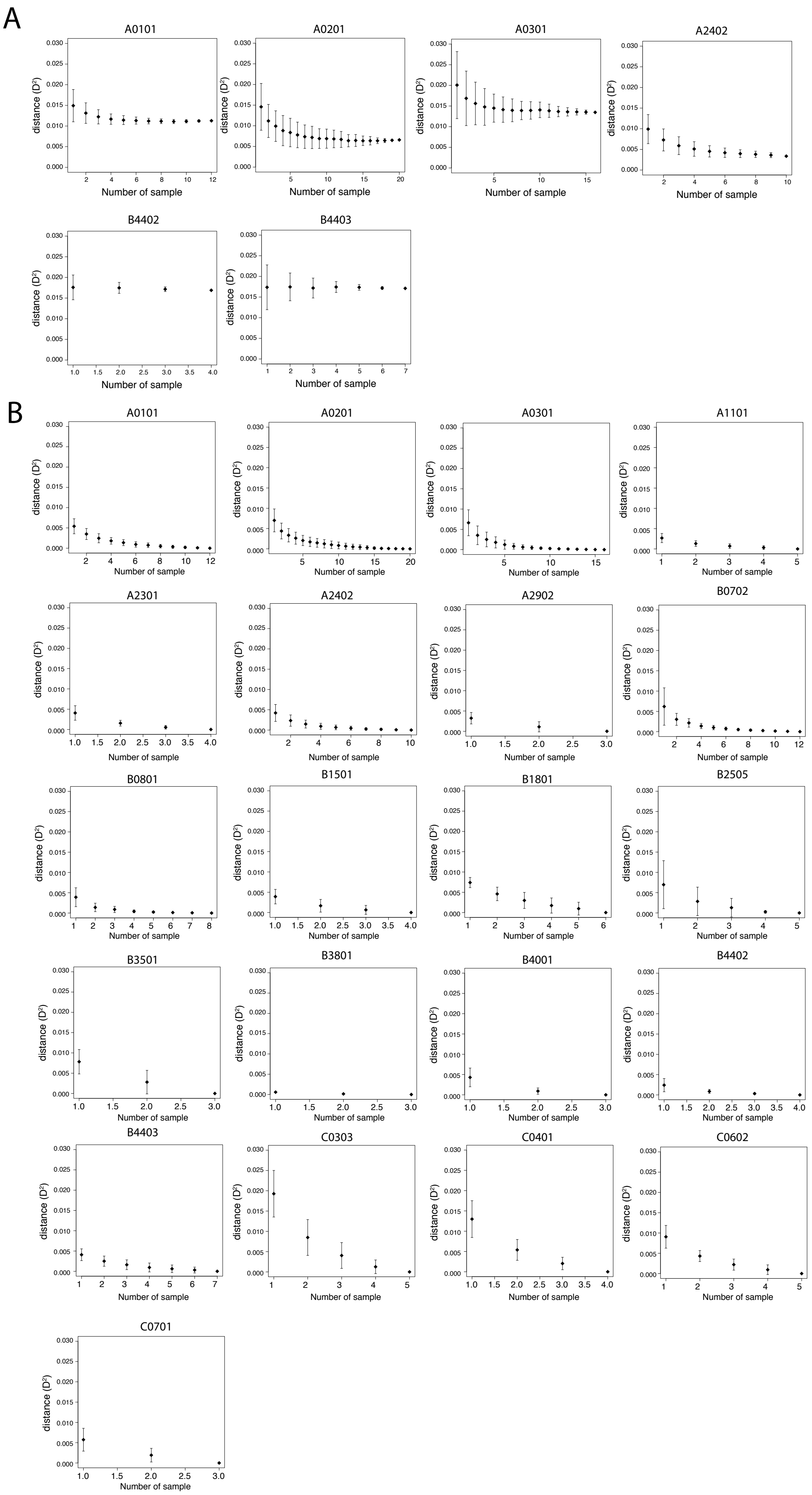

A
A0101
A0201
A0301
A2402
B4402
B4403
B
A0101
A0201
A0301
A1101
A2301
A2402
A2902
B0702
B0801
B1501
B1801
B2505
B3501
B3801
B4001
B4402
B4403
C0303
C0401
C0602
C0701
distance (D^2)
Number of sample

Fig F: (a) Distance between the motifs predicted in our work and those derived from mono-allelic cell lines [2], as a function of the number of samples that are considered (error bars correspond to 100 random choices). The six alleles were selected as those with motifs detected in more than 3 samples in our dataset of pooled HLA peptidomics data and present in the mono-allelic cell lines of [2]. Of note, some small contaminations or batch effects are expected even in mono-allelic cell lines and we did not expect distances to fall exactly to zero. (b) Same as in A, but comparing with the motifs derived from all samples, for all alleles present in at least three samples. By construction all distances fall to 0. The most important message of this figure is that all distances (even when considering only one sample) are very small, and much smaller than the 0.078 threshold used to determine two motifs as 'similar'.

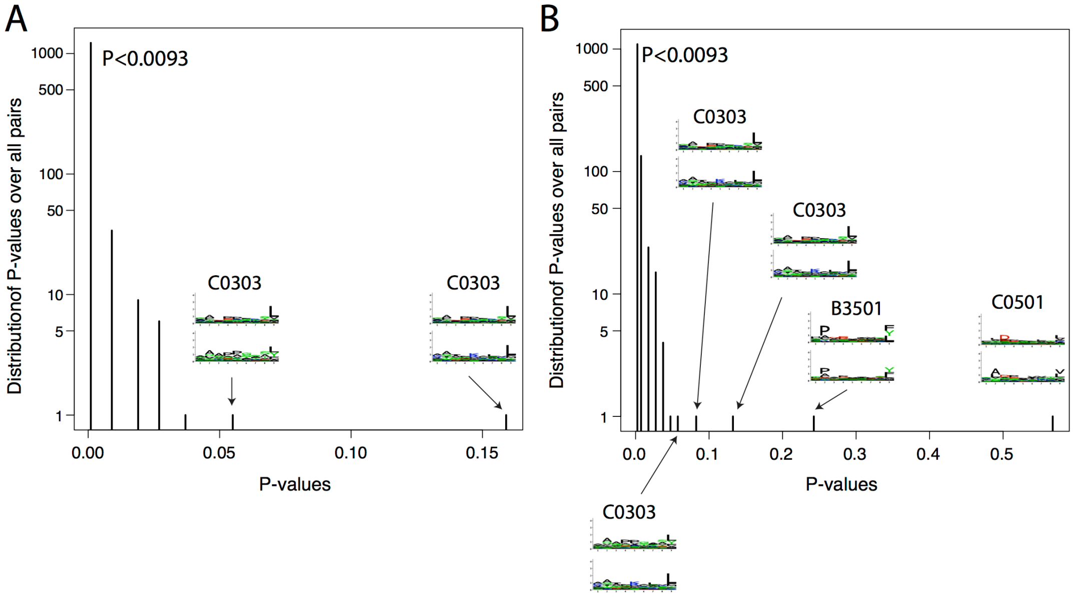

Fig G: Distribution of P-values of similarities over all pairs of motifs annotated to the same alleles. Empirical P-values were computed by comparing for each pair of motif (m_i, m_j) the similarity between m_i and the set of known HLA-I motifs, excluding the one to which m_i had been annotated (107 motifs in total, see Methods). In (a), the Euclidean distance was used and in (b) the BLiC score of ref. [3]. The first bar of the graphs shows the number of cases where the similarity was higher than to any of the known HLA-I motifs used for estimating these empirical P-values ($P < 1/107 = 0.0093$). The very few pairs of motifs that do not pass the statistical threshold are shown explicitly, as well as the allele to which they had been assigned.

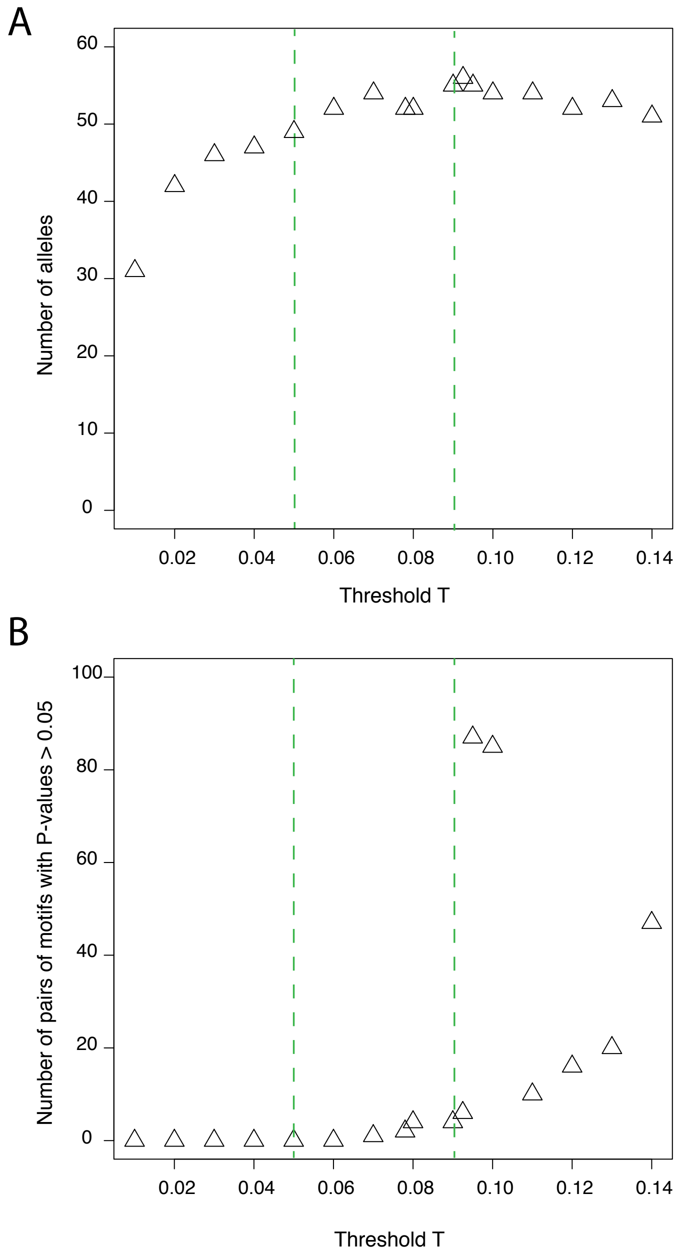

Fig H: Effect of different thresholds T for the Euclidean distance. (a) Number of HLA-I alleles with at least one motif annotated to them as a function of T. (b) Number of pairs of motifs annotated to the same allele with similarity P-values (based on Euclidean distance) larger than 0.05, as a function of T. The green bars indicate a possible range of values of T that leads to similar number of alleles being detected and few pairs of motifs with P-values > 0.05. The non-linearity can be expected since our algorithm is an optimization procedure.

HLA alleles	Predicted logo	Known ligands
HLA-A25:01	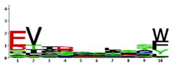	*no ligands*
HLA-B13:02	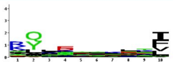	GQFKDIITKV YILGADPLRV
HLA-B38:01	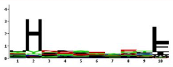	*no ligands*
HLA-B39:06	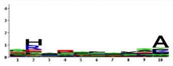	*no ligands*
HLA-B56:01	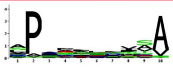	*no ligands*
HLA-B73:01	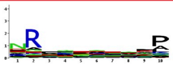	*no ligands*

Fig I: New HLA-I binding motifs identified for 10-mers across all HLA peptidomics datasets.

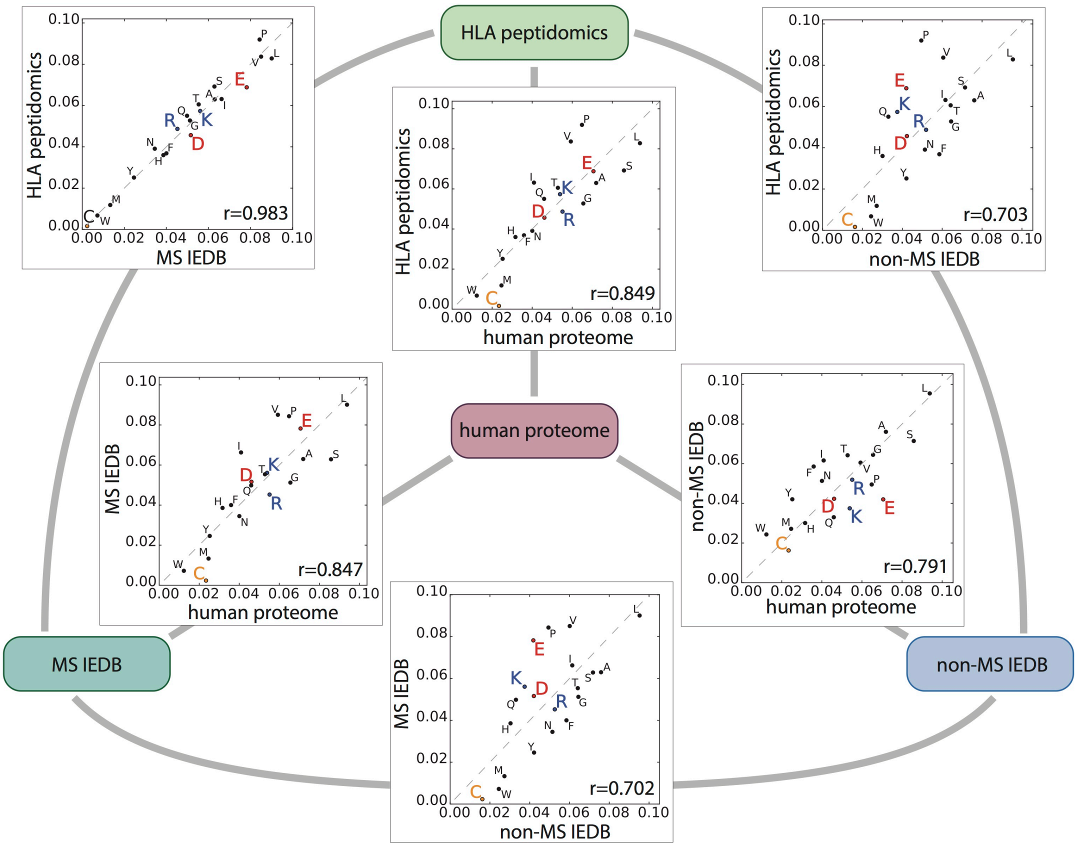

Fig J: Comparison between amino acid frequencies at positions P4 to P7 in HLA peptidomics data from unmodified cell lines or tissue samples, MS IEDB data, non-MS IEDB data, and the human proteome. Only alleles with at least 100 ligands in our dataset, IEDB non-MS and IEDB MS were used (marked with * in Fig D).

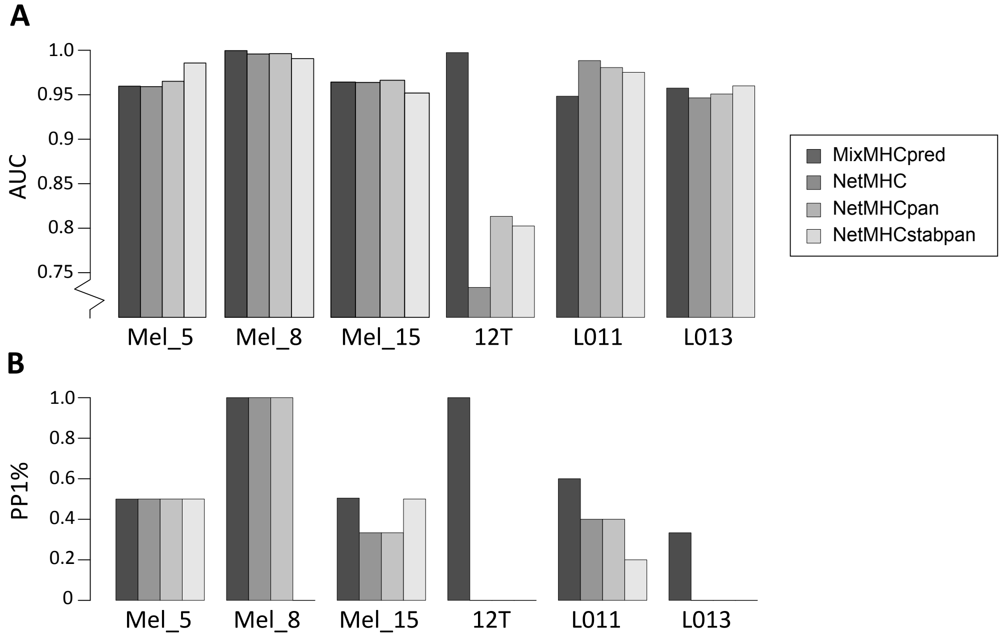

Fig K: Comparison between our predictor (MixMHCpred) and existing tools when excluding endogeneous HLA-I ligands from the sample in which mutated neo-antigens had been identified (same comparison as in Fig 4B-C, other benchmarkings remain the same).

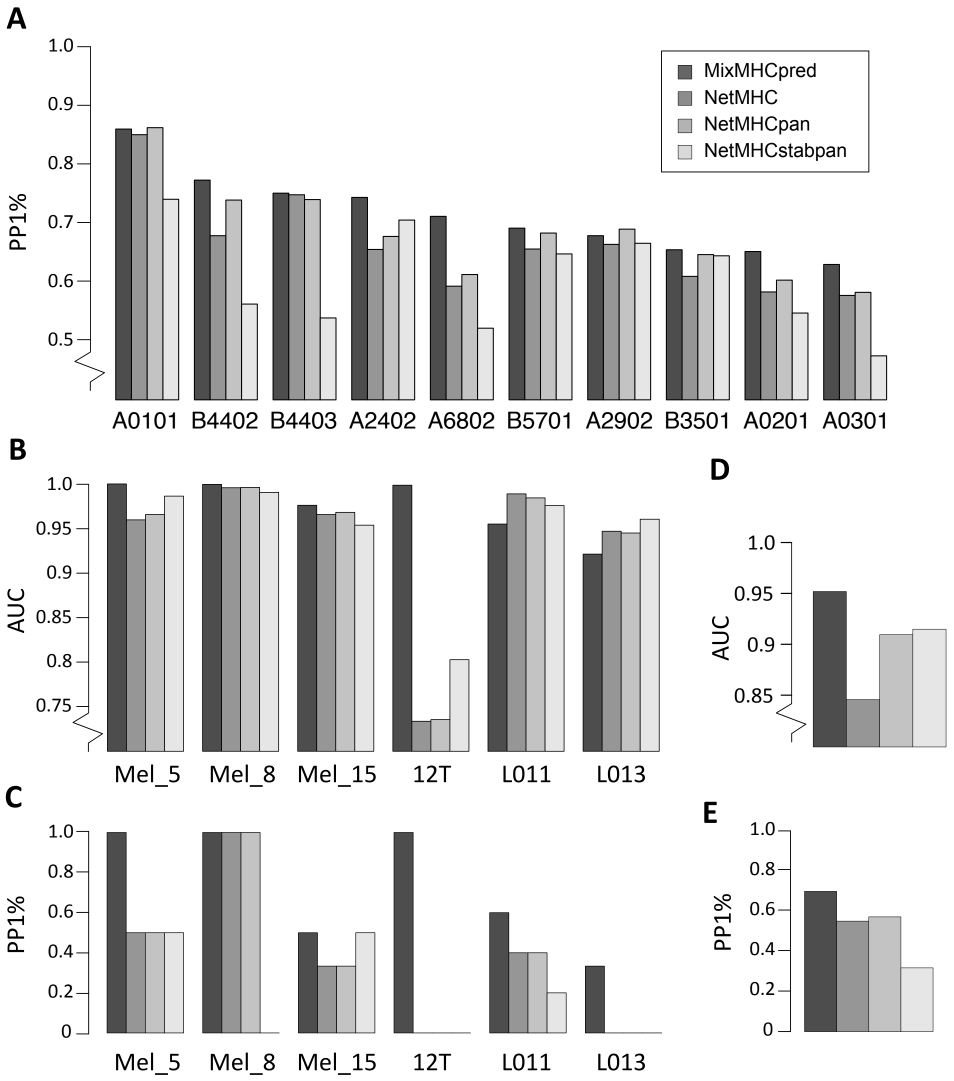

Fig L: Comparison between our predictor (MixMHCpred) and existing tools when adding 5% of noise in the pooled HLA peptidomics data used to train our predictor.

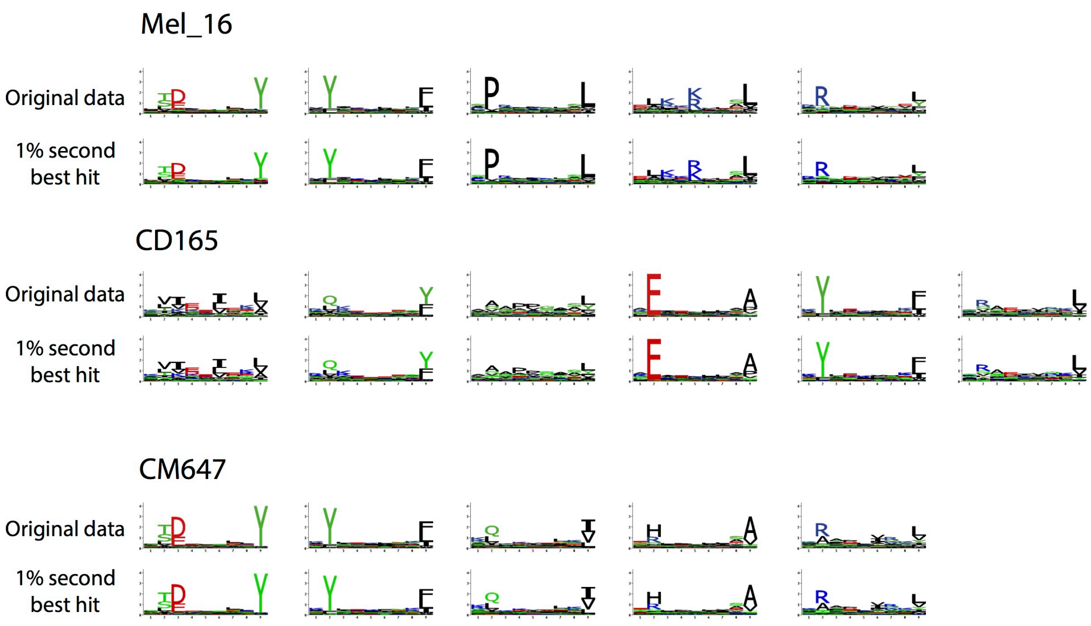

Fig M: Comparison of the deconvoluted motifs for the three samples shown in Fig 1, when using the second best hit in spectral searches for 1% of the peptides.

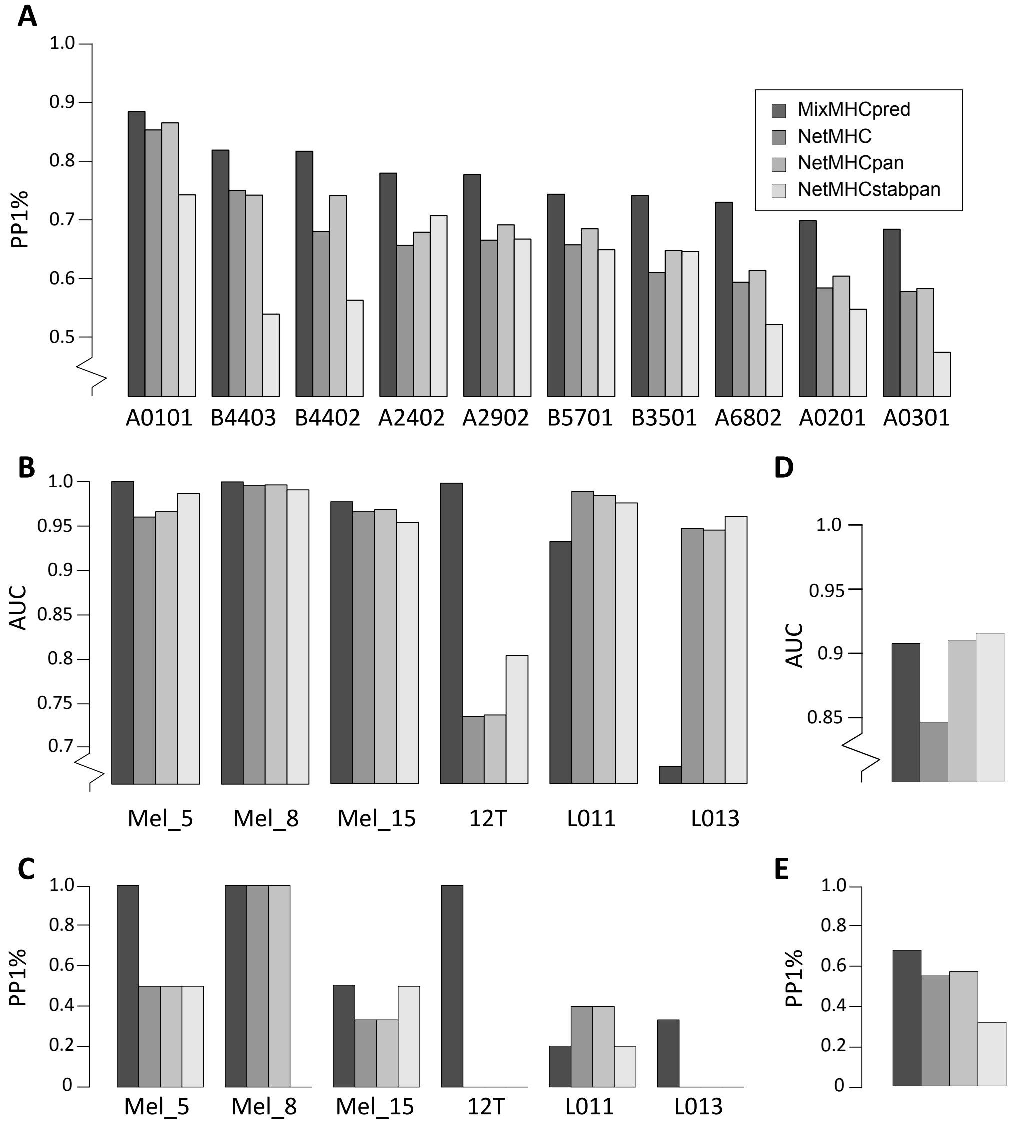

Fig N: Performance of predictors when using amino acid frequencies from the human proteome (instead of those found at non-anchor residues) in MixMHCpred (same data as in Fig 4). In this case, cysteine containing peptides are poorly predicted, which increases the performance of MixMHCpred on elution data (mainly panel A), but decreases the performance on other datasets (especially L011 and L013).

HLA alleles	Binding motif	Amino acid at position 90
HLA-A02:20	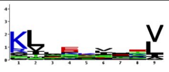	asparagine
HLA-A03:01	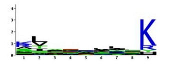	asparagine
HLA-A03:19	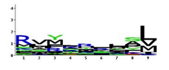	asparagine
HLA-A30:01	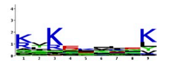	asparagine
HLA-A30:04	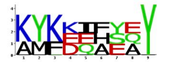	asparagine
HLA-A31:01	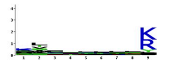	asparagine
HLA-A32:01	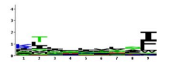	asparagine
HLA-A32:07	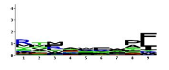	asparagine
HLA-B13:01	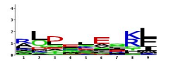	isoleucine
HLA-B13:02	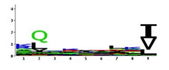	isoleucine
HLA-B15:01	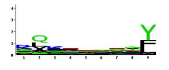	isoleucine
HLA-B15:42	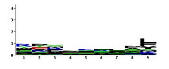	isoleucine

HLA alleles	Binding motif	Amino acid at position 90
HLA-B27:10	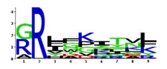	isoleucine
HLA-B27:20	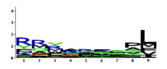	isoleucine
HLA-B40:13	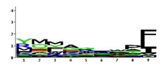	isoleucine
HLA-B41:01	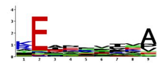	isoleucine
HLA-B41:02	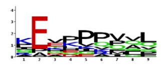	isoleucine
HLA-B42:01	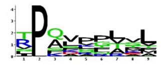	isoleucine
HLA-B47:01	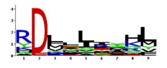	isoleucine
HLA-B48:01	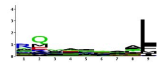	isoleucine
HLA-B57:01	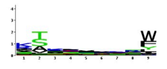	asparagine
HLA-B57:02	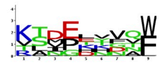	asparagine
HLA-B57:03	X-ray structure with `KAFSPEVI`	asparagine
HLA-C15:02	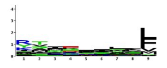	asparagine

Fig O: Binding motifs and amino acid at position 66 for HLA-I alleles showing some preference for charged amino acids (R or K) at P1.

A

HLA alleles	Binding motif
HLA-B15:09	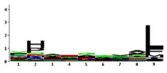
HLA-B15:10	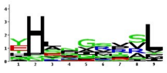
HLA-B15:18	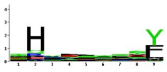
HLA-B38:01	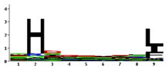
HLA-B39:01	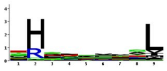
HLA-B39:05	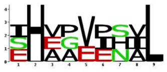
HLA-B39:06	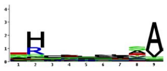
HLA-B39:09	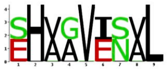

B

HLA alleles	Binding motif
HLA-B14:01	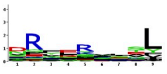
HLA-B14:02	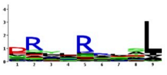
HLA-B14:03	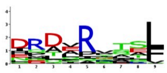

Fig P: (a) Binding motifs for HLA-I alleles showing preference for histidine at P2. (b) Binding motifs for HLA-B14 alleles displaying the same P2 binding site as HLA-B15:18.

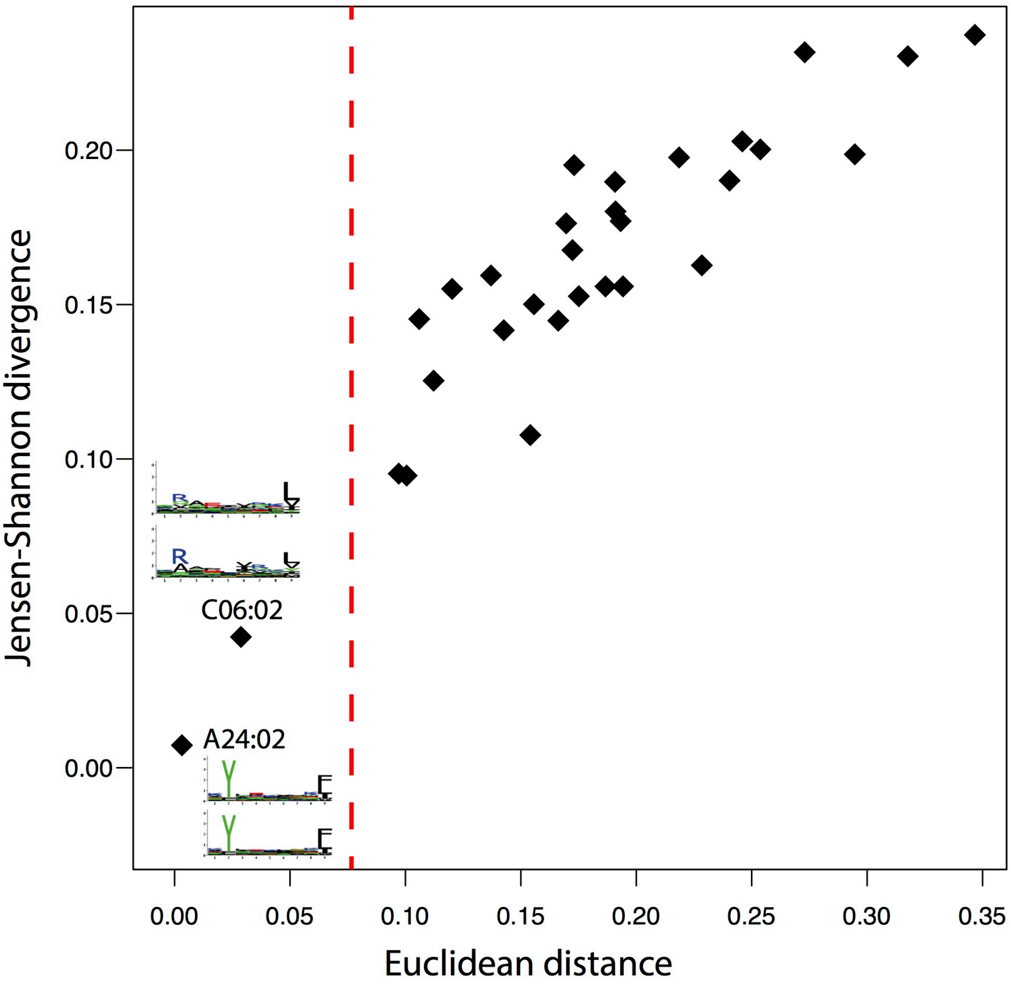

Fig Q: Comparison of the motif similarity values using Euclidean distance and Jensen-Shannon divergence (average of Jensen-Shannon divergence over all 9 positions) for all 5x6 pairs of motifs between two last samples (CD165 and CM467) shown in Fig 1. The red line shows the cut-off on Euclidean distance used in this work.

Table A: HLA peptidomics datasets analyzed in this work. Columns 2 and 3 show the number of unique peptides in each dataset. The first ten samples have been generated for this work.

Samples	9-mers	10-mers	HLA-A	HLA-A	HLA-B	HLA-B	HLA-C	HLA-C
CD165	3799	847	A02:05	A24:02	B15:01	B50:01	C03:03	C06:02
CM467	5279	921	A01:01	A24:02	B13:02	B39:06	C06:02	C12:03
GD149	5960	2063	A01:01	A24:02	B38:01	B44:03	C06:02	C12:03
MD155	3089	493	A02:01	A24:02	B15:01	B18:01	C03:03	C07:01
PD42	1392	450	A02:06	A24:02	B07:02	B55:01	C01:02	C07:02
RA957	6652	1944	A02:20	A68:01	B35:03	B39:01	C04:01	C07:02
TIL1	4012	711	A02:01	B18:01	B38:01		C05:01	
TIL3	5896	1290	A01:01	A23:01	B07:02	B15:01	C12:03	C14:02
Apher1	3527	1273	A03:01	A29:02	B44:02	B44:03	C12:03	C16:01
Apher6	1138	376	A02:01	A03:01	B07:02		C07:02	
Mel15 [1]	11094	4865	A03:01	A68:01	B27:05	B35:03	C02:02	C04:01
Mel16 [1]	7460	1769	A01:01	A24:02	B07:02	B08:01	C07:01	C07:02
Mel12 [1]	2467	417	A01:01		B08:01		C07:01	
Mel8 [1]	3408	1283	A01:01	A03:01	B07:02	B08:01	C07:01	C07:02
Mel5 [1]	3108	750	A01:01	A25:01	B08:01	B18:01		
Fibroblast [4]	3492	859	A03:01	A23:01	B08:01	B15:18	C07:02	C07:04
HCC1143 [4]	1989	284	A31:01	B35:08	B37:01	C04:01	C06:02	
HCC1937 [4]	2926	1065	A23:01	A24:02	B07:02	B40:01	C03:04	C07:02
HCT116 [4]	2665	737	A01:01	A02:01	B45:01	B18:01	C05:01	C07:01
JY [4]	1740	483	A02:01		B07:02		C07:02	
Bcell [5]	5974	2521	A01:01	A03:01	B07:02	B27:05	C02:02	C07:01
Mel-624 [6]	1403	425	A02:01	A03:01	B07:02	B14:01	C07:02	C08:01
SK-Mel-5 [6]	1845	618	A02:01	A11:01	B40:01		C03:03	
HEK293 [7]	2477	1221	A03:01		B07:02		C07:02	
MAVER_1 [7]	4912	1423	A24:02	A26:01	B38:01	B44:02	C05:01	C12:03
HL_60 [7]	3308	1437	A01:01		B57:01		C06:02	
RPMI8226 [7]	3237	647	A30:01	A68:02	B15:03	B15:10	C02:10	C03:04
THP_1 [7]	4085	656	A02:01	A24:02	B15:11	B35:01	C03:03	
CA46 [7]	2063	414	A26:03		B27:04		C12:02	
LNT-229 [8]	5202	2191	A03:01		B35:01		C04:01	
T98G [8]	6294	1413	A02:01		B39:06		C07:02	
U-87 [8]	6992	2126	A02:01		B44:02		C05:01	
pat_AC2 [9]	920	267	A03:01	A32:01	B27:05	B45:01		
pat_C [9]	1423	641	A02:01	A03:01	B07:02		C07:02	

pat_CELG [9]	2068	664	A02:01	A24:02	B15:01	B73:01	C03:03	C15:05
pat_CP2 [9]	1055	376	A11:01		B14:02	B44:02		
pat_FL [9]	2127	873	A03:01	A11:01	B44:03	B50:01		
pat_J [9]	1215	551	A02:01	A03:01	B07:02		C07:02	
pat_JPB3 [9]	1009	487	A02:01	A11:01	B27:05	B56:01		
pat_JT2 [9]	846	287	A11:01		B18:03	B35:01		
pat_M [9]	1523	574	A03:01	A29:02	B08:01	B44:03	C07:01	C16:01
pat_MA [9]	1998	808	A02:01	A29:02	B44:03	B57:01	C07:01	C16:01
pat_ML [9]	1580	811	A02:01	A11:01	B40:01	B44:03		
pat_NS2 [9]	435	91	A02:01		B13:02	B41:01		
pat_NT [9]	1522	252	A01:01	A32:01				
pat_PF1 [9]	2484	1049	A01:01	A02:01	B07:02	B44:03	C07:02	C16:01
pat_R [9]	1406	549	A03:01	A29:02	B08:01	B44:03	C07:01	C16:01
pat_RT [9]	1425	333	A01:01	A02:01	B18:01	B39:24	C05:01	C07:01
pat_SR [9]	1642	505	A02:01	A23:01	B18:01	B44:03		
pat_ST [9]	771	283	A03:01	A24:02	B07:02	B27:05		

Table B: Amino acid frequencies at non-anchor positions (P4 to P7) in HLA-I ligands from pooled HLA peptidomics data.

A	0.060782
C	0.001453
D	0.045653
E	0.073598
F	0.035112
G	0.049635
H	0.037461
I	0.06487
K	0.059036
L	0.083318
M	0.011609
N	0.039796
P	0.090261
Q	0.056825
R	0.050559
S	0.066348
T	0.059356
V	0.083667
W	0.006434
Y	0.024228

Table C: Ranking of the neo-antigens identified in two lung cancer samples [10]. Column 4 shows the ranking based on our predictor. Column 6-8 show the ranking based on NetMHC[11], NetMHCpan[12] and NetMHCstabpan[13].

Sample	Sequence	Protein	Muta-tion	Rank	Net-MHC	Net-MHC-pan	Net-MHC-stabpan	# Candi-dates
L011	SVTNEFC**L**K	DOCK10	R1149L	**13**	26	21	9	7414
L011	GTSAPRKKK	XIRP2	Q2514K	**58**	213	281	110	7414
L011	FAFQE**Y**DSF	MTFR2	D326Y	65	**6**	10	296	7414
L011	RSMRTVYGL**F**	OR2A2	C317F	712	**83**	136	221	7414
L011	GPEELGLP**M**	SOHLH2	L159M	1070	**109**	158	292	7414
L013	ALQSRLQAL	CACNA1I	R464L	**3**	151	178	118	2700
L013	KVCCCQILL	OR4P4	W300C	168	224	**153**	**153**	2700
L013	YSNYY**C**GLRY	KRTAP20-2	G9C	177	63	124	**58**	2700

Table D: List of cancer testis antigens with epitopes and HLA restriction information from the CTDatabase [14].

**Protein**	**Epitope**	**Allele**	**Protein**	**Epitope**	**Allele**
BAGE	AARAVFLAL	C16:01	MAGEA12	EGDCAPEEK	C07:01
CCDC110	NYNNFYRFL	A24:02	MAGEA2	YLQLVFGIEV	A02:01
CCDC110	EYSKECLKEF	A24:02	MAGEA2	EYLQLVFGI	A24:02
CCDC110	EYLSLSDKI	A24:02	MAGEA2	EGDCAPEEK	C07:01
CTAG1A	SLLMWITQC	A02:01	MAGEA3	EVDPIGHLY	A01:01
CTAG1A	ASGPGGGAPR	A02:01	MAGEA3	FLWGPRALV	A02:01
CTAG1A	MPFATPMEA	B35:01	MAGEA3	KVAELVHFL	A02:01
CTAG1A	MPFATPMEA	B51:01	MAGEA3	TFPDLESEF	A24:02
CTAG1A	ARGPESRLL	C06:02	MAGEA3	VAELVHFLL	A24:02
CTAG1A	LAMPFATPM	C03:03	MAGEA3	MEVDPIGHLY	B18:01
CTAG2	ELVRRILSR	A68:01	MAGEA3	EVDPIGHLY	B35:01
CT83	RQKRILVNL	B15:01	MAGEA3	AELVHFLLL	B40:01
MAGEA1	EADPTGHSY	A01:01	MAGEA3	MEVDPIGHLY	B44:02
MAGEA1	KVLEYVIKV	A02:01	MAGEA3	EGDCAPEEK	C07:01
MAGEA1	SLFRAVITK	A03:01	MAGEA4	EVDPASNTY	A01:01
MAGEA1	EVYDGREHSA	A68:02	MAGEA4	GVYDGREHTV	A02:01
MAGEA1	RVRFFFPSL	B07:02	MAGEA4	NYKRCFPVI	A24:02
MAGEA1	EADPTGHSY	B35:01	MAGEA6	EVDPIGHVY	B35:01
MAGEA1	SAYGEPRKL	C03:03	MAGEA6	EGDCAPEEK	C07:01
MAGEA1	SAYGEPRKL	C16:01	MAGEA6	ISGGPRISY	C16:01
MAGEA10	GLYDGMEHL	A02:01	MAGEA9	ALSVMGVYV	A02:01
MAGEA12	FLWGPRALV	A02:01	SAGE1	LYATVIHDI	A24:02
MAGEA12	FLWGPRALV	C07:01	SSX2	KASEKIFYV	A02:01

References

1. Bassani-Sternberg M, Bräunlein E, Klar R, Engleitner T, Sinitcyn P, Audehm S, et al. Direct identification of clinically relevant neoepitopes presented on native human melanoma tissue by mass spectrometry. Nat Commun. Nature Publishing Group; 2016;7: 13404. doi:10.1038/ncomms13404

2. Abelin JG, Keskin DB, Sarkizova S, Hartigan CR, Zhang W, Sidney J, et al. Mass Spectrometry Profiling of HLA-Associated Peptidomes in Mono-allelic Cells Enables More Accurate Epitope Prediction. Immunity. 2017;46: 315–326. doi:10.1016/j.immuni.2017.02.007

3. Habib N, Kaplan T, Margalit H, Friedman N. A novel Bayesian DNA motif comparison method for clustering and retrieval. Fraenkel E, editor. PLoS Comput Biol. 2008;4: e1000010. doi:10.1371/journal.pcbi.1000010

4. Bassani-Sternberg M, Pletscher-Frankild S, Jensen LJ, Mann M. Mass spectrometry of human leukocyte antigen class I peptidomes reveals strong effects of protein abundance and turnover on antigen presentation. Mol Cell Proteomics. 2015;14: 658–673. doi:10.1074/mcp.M114.042812

5. Mommen GPM, Frese CK, Meiring HD, van Gaans-van den Brink J, de Jong APJM, van Els CACM, et al. Expanding the detectable HLA peptide repertoire using electron-transfer/higher-energy collision dissociation (EThcD). Proc Natl Acad Sci USA. National Acad Sciences; 2014;111: 4507–4512. doi:10.1073/pnas.1321458111

6. Gloger A, Ritz D, Fugmann T, Neri D. Mass spectrometric analysis of the HLA class I peptidome of melanoma cell lines as a promising tool for the identification of putative tumor-associated HLA epitopes. Cancer Immunol Immunother. 2016;65: 1377–1393. doi:10.1007/s00262-016-1897-3

7. Ritz D, Gloger A, Weide B, Garbe C, Neri D, Fugmann T. High-sensitivity HLA class I peptidome analysis enables a precise definition of peptide motifs and the identification of peptides from cell lines and patients' sera. Proteomics. 2016;: n/a–n/a. doi:10.1002/pmic.201500445

8. Shraibman B, Kadosh DM, Barnea E, Admon A. Human Leukocyte Antigen (HLA) Peptides Derived from Tumor Antigens Induced by Inhibition of DNA Methylation for Development of Drug-facilitated Immunotherapy. Mol Cell Proteomics. American Society for Biochemistry and Molecular Biology; 2016;15: 3058–3070. doi:10.1074/mcp.M116.060350

9. Pearson H, Daouda T, Granados DP, Durette C, Bonneil E, Courcelles M, et al. MHC class I-associated peptides derive from selective regions of the human genome. J Clin Invest. American Society for Clinical Investigation; 2016;126. doi:10.1172/JCI88590

10. Bentzen AK, Marquard AM, Lyngaa R, Saini SK, Ramskov S, Donia M, et al. Large-scale detection of antigen-specific T cells using peptide-MHC-I multimers labeled with DNA barcodes. Nat Biotechnol. Nature Research;

2016;34: 1037–1045. doi:10.1038/nbt.3662

11. Andreatta M, Nielsen M. Gapped sequence alignment using artificial neural networks: application to the MHC class I system. Bioinformatics. 2015. doi:10.1093/bioinformatics/btv639

12. Nielsen M, Andreatta M. NetMHCpan-3.0; improved prediction of binding to MHC class I molecules integrating information from multiple receptor and peptide length datasets. Genome Med. BioMed Central; 2016;8: 33. doi:10.1186/s13073-016-0288-x

13. Rasmussen M, Fenoy E, Harndahl M, Kristensen AB, Nielsen IK, Nielsen M, et al. Pan-Specific Prediction of Peptide-MHC Class I Complex Stability, a Correlate of T Cell Immunogenicity. J Immunol. American Association of Immunologists; 2016;197: 1517–1524. doi:10.4049/jimmunol.1600582

14. Almeida LG, Sakabe NJ, deOliveira AR, Silva MCC, Mundstein AS, Cohen T, et al. CTdatabase: a knowledge-base of high-throughput and curated data on cancer-testis antigens. Nucleic Acids Res. 2009;37: D816–9. doi:10.1093/nar/gkn673
